# Evaluation and Modelling of the Performance of an Automated SARS-CoV-2 Antigen Assay According to Sample Type, Target Population and Epidemic Trends

**DOI:** 10.3390/diagnostics12020447

**Published:** 2022-02-09

**Authors:** Nicolas Yin, Cyril Debuysschere, Valery Daubie, Marc Hildebrand, Charlotte Martin, Sonja Curac, Fanny Ponthieux, Marie-Christine Payen, Olivier Vandenberg, Marie Hallin

**Affiliations:** 1 Department of Microbiology, Laboratoire Hospitalier Universitaire de Bruxelles-Universitair Laboratorium Brussel (LHUB-ULB), Université Libre de Bruxelles (ULB), 1000 Brussels, Belgium; cyril.debuysschere@lhub-ulb.be (C.D.); valery.daubie@lhub-ulb.be (V.D.); fanny.ponthieux@lhub-ulb.be (F.P.); marie.hallin@ulb.be (M.H.); 2 Department of Internal Medicine, Erasme University Hospital, Université Libre de Bruxelles (ULB), 1070 Brussels, Belgium; marc.hildebrand@erasme.ulb.ac.be; 3 Department of Infectious Diseases, Saint-Pierre University Hospital, Université Libre de Bruxelles (ULB), 1000 Brussels, Belgium; charlotte.martin@stpierre-bru.be (C.M.); marie-christine.payen@stpierre-bru.be (M.-C.P.); 4 Emergency Department, Erasme University Hospital, Université Libre de Bruxelles (ULB), 1070 Brussels, Belgium; sonja.curac@erasme.ulb.ac.be; 5 Centre for Environmental Health and Occupational Health, School of Public Health, Université Libre de Bruxelles (ULB), 1050 Brussels, Belgium; olivier.vandenberg@lhub-ulb.be; 6 Clinical Research and Innovation Unit, Laboratoire Hospitalier Universitaire de Bruxelles-Universitair Laboratorium Brussel (LHUB-ULB), Université Libre de Bruxelles (ULB), 1000 Brussels, Belgium; 7 Division of Infection and Immunity, Faculty of Medical Sciences, University College London, London WC1E 6BT, UK

**Keywords:** SARS-CoV-2, COVID-19, model, diagnostic, test, assay

## Abstract

The Lumipulse^®^ *G* SARS-CoV-2 Ag assay performance was evaluated on prospectively collected saliva and nasopharyngeal swabs (NPS) of recently ill in- and outpatients and according to the estimated viral load. Performances were calculated using RT-PCR positive NPS from patients with symptoms ≤ 7 days and RT-PCR negative NPS as gold standard. In addition, non-selected positive NPS were analyzed to assess the performances on various viral loads. This assay yielded a sensitivity of 93.1% on NPS and 71.4% on saliva for recently ill patients. For NPS with a viral load > 10^3^ RNA copies/mL, sensitivity was 96.4%. A model established on our daily routine showed fluctuations of the performances depending on the epidemic trends but an overall good negative predictive value. Lumipulse^®^ *G* SARS-CoV-2 assay yielded good performance for an automated antigen detection assay on NPS. Using it for the detection of recently ill patients or to screen high-risk patients could be an interesting alternative to the more expensive RT-PCR.

## 1. Introduction

The Lumipulse^®^ *G* SARS-CoV-2 Ag assay (Fujirebio, Tokyo, Japan) is an automated chemiluminescence enzyme immunoassay (CLEIA) allowing SARS-CoV-2 antigen quantification. Its throughput is between 60 to 120 samples per hour depending on the instrument used (Lumipulse^®^ G600II or G1200) and it can be performed on both universal transport medium (UTM) preserved nasopharyngeal swabs (NPS) and saliva. Saliva sample use could indeed improve the comfort of patients, and decrease the needs in terms of swabs and transport media [1,2]. The World Health Organization (WHO) recommends a ≥80% sensitivity and ≥97% specificity for antigen detection test but without providing any specific setting [3]. The European Centre for Disease Control agrees with the WHO but advocates for higher performance (≥90% sensitivity and >98% specificity) for samples taken within 5 days from symptom onset (DSO) or seven days from exposure [4]. While global performance of the Lumipulse^®^ *G* SARS-CoV-2 Ag assay was assessed previously [5,6,7,8], performance data are still missing regarding saliva samples for this latter specific population. Furthermore, existing studies [5,6,7,8] did not use the pretreatment step recommended now by the manufacturer for viral deactivation and sample fluidification. Adding an in-house inactivation step does improve safety but can also decrease the test sensitivity [9]. Here, we evaluated the performance of the Lumipulse^®^ *G* SARS-CoV-2 assay using the manufacturer’s sample extraction solution (SES) pretreatment on both NPS and saliva and assessed its performance with regards to the target population and the viral load. In a second step, we used the semi-quantitative PCR results from our daily routine to model the influence of the epidemic curve on the overall performance of this assay.

## 2. Materials and Methods

### 2.1. Study Design, Population and Sample Collection

Residual UTMs of patients’ NPS were selected on the basis of SARS-CoV-2 daily routine RT-PCR results. In particular, and as required for the CE-IVD certification, a minimum of 300 SARS-CoV-2 negative RT-PCR samples from outpatients, 100 SARS-CoV-2 negative RT-PCR samples from inpatients and 100 SARS-CoV-2 positive RT-PCR samples from patients with maximum 7 DSO were included. In addition, residuals of consecutive available positive samples that did not meet the above selection criteria (i.e., either taken more than 7 DSO or for which DSO was not known) were included to better assess the limit of detection by increasing the number of positive samples and broadening the range of antigen concentration tested. All these samples were stored at 4 °C and analyzed within 2 days after the RT-PCR was performed.

Saliva samples were collected from patients prospectively enrolled in two settings: outpatients at the consultation or at the sampling center with a prescription of a SARS-CoV-2 PCR test on NPS as well as inpatients with a positive SARS-CoV-2 RT-PCR test on NPS for less than 2 days. The NPS was paired with the saliva sample when available.

### 2.2. SARS-CoV-2 RT-PCR

SARS-CoV-2 RT-PCR on NPS was considered as the gold standard. If the NPS was not available anymore to perform the assay, the RT-PCR performed on saliva was considered as a proxy of the gold standard.

The RT-PCR was performed using the Alinity *m* SARS-CoV-2 assay (Abbott Molecular, Des Plaines, IL, USA). The two target sequences (in the RdRp and the N genes) are detected using the same fluorophore. A 4-fold dilution was performed on saliva samples before extraction to reach the volume of 500 µL needed for this assay. Cycle threshold (Ct) values were plotted with standards provided by the Belgian national reference center following recommendations by Sciensano to provide semi-quantitative results [10]. The correspondence table between Ct values and viral load estimation is summarized in Table 1.

### 2.3. SARS-CoV-2 Antigen Quantification

Antigen quantification was performed using the Lumipulse^®^ *G* SARS-CoV-2 Ag assay, expressing the dosage in pg/mL. Viral inactivation was performed using the sample extraction solution (SES) by diluting 4 parts of UTM with 1 part of SES or 1 part of saliva with 1 part of SES. A 30 min incubation at room temperature was performed before centrifugation at 2000× *g* for 5 min. Lumipulse^®^ SARS-CoV-2 antigen testing was performed on a Lumipulse^®^ G600II instrument according to the manufacturer’s instruction. To obtain a binary qualitative result (negative/positive), a positivity threshold was determined using the positive samples from patients with ≤7 DSO and all the negative samples using the following criteria: highest possible Youden’s index, specificity >98% and a minimum of 0.6 pg/mL (instrument quantification limit). Samples presenting a dosage below this threshold were considered as negative.

### 2.4. SARS-CoV-2 Variant Determination

As a part of the Belgian national surveillance program, all the strong and very strong available positive samples (see Table 1) were prospectively sequenced at the time of the experimentation using the COVIDSeq kit on NextSeq (Illumina, San Diego, CA, USA). Hence, they represent the variants circulating at the time of the study in Belgium.

### 2.5. Model

All the available positive RT-PCR results of our clinical laboratory were semi-quantified according to their Ct values regardless of their indication (symptoms, screening…). Daily overall sensitivity (Se), positive predictive value (PPV) and negative predictive value (NPV) were estimated according to the performance of the automated antigen quantification for each semi-quantitative RT-PCR category. To minimize day-to-day and holiday-related fluctuations, data were computed from 1 May 2020 to 30 October 2021 using a moving average of 14 days (hereafter referred as “14-day Se”, “14-day PPV” and “14-day NPV”).

### 2.6. Statistical Analyses

Statistical analyses and receiver operating characteristic (ROC) curves were performed using Analyse-it^®^ for Microsoft Excel v5.30.4.

### 2.7. Ethical Approval

The Erasme University Hospital Ethics Committee approved and reviewed the comparative performance evaluation study on saliva and NPS. The Ethics Committee of the Saint-Pierre Hospital waived ethical approval for the use of residual human body material for evaluation purpose.

## 3. Results

### 3.1. Population

In total, 632 patients were included with 605 NPS and 144 saliva of which 117 were paired samples. The details of the repartition between in- and outpatients for negative samples and DSO for positive samples are summarized in Table 2.

### 3.2. Threshold Determination

#### 3.2.1. NPS

**Figure 1:**
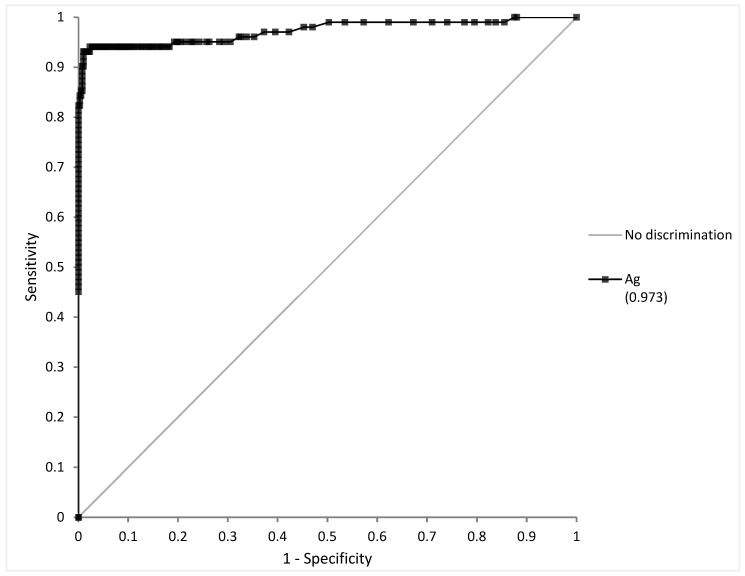
Receiver Operating Curve (ROC) analysis of antigen quantification (Ag) on nasopharyngeal swabs (NPS) vs. RT-PCR on NPS as gold standard.

A total of 502 samples were selected: 102 positive samples with ≤7 DSO and 400 negative samples. ROC curve analysis yielded an area under the curve (AUC) at 0.973 ± 0.023 (Figure 1). The highest Youden index was at a threshold of 2.47 pg/mL (sensitivity 93.1%, specificity 99.0%). Analytical performance and their confidence interval at this threshold are summarized in Table 3.

#### 3.2.2. Saliva

**Figure 2:**
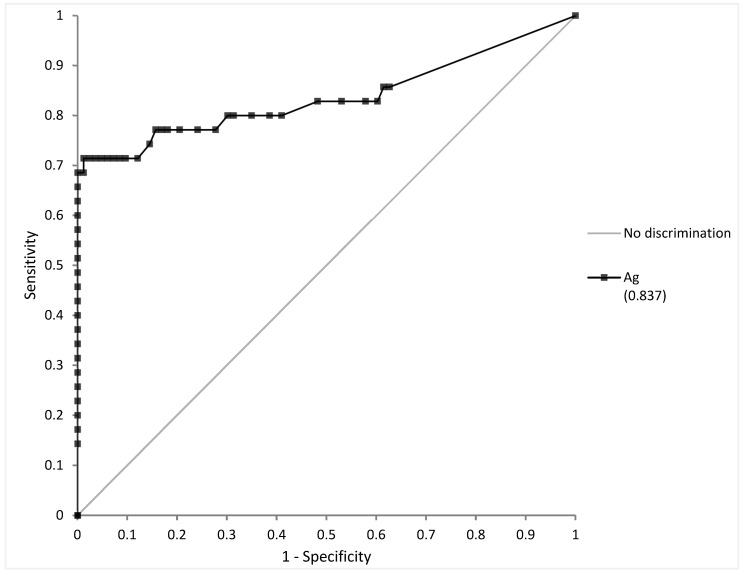
Receiver Operating Curve (ROC) analysis of antigen quantification (Ag) on saliva vs. RT-PCR on nasopharyngeal swabs (NPS) as gold standard.

In total, 118 samples were selected: 35 positive samples with ≤7 DSO and 83 negative samples. ROC curve analysis yielded an area under the curve (AUC) at 0.973 ± 0.023 (Figure 2). The highest Youden index was at a threshold of 0.55 pg/mL (sensitivity 71.4%, specificity 98.8%). To respect the quantification limit, the positivity threshold was set at 0.6 pg/mL. Analytical performance and their confidence interval at this threshold are summarized in Table 3.

### 3.3. Detection Limit Assessment

The sensitivity of antigen quantification was better for higher estimated viral loads with an overall sensitivity of 96.4% on NPS and of 69.6% on saliva when excluding the low positive samples (estimated viral load < 10^3^ RNA copies/mL, Ct > 29.9), whereas the sensitivity for the low positive samples was 10.5% on NPS. The detailed performance at four different levels of RT-PCR semi-quantification on NPS is summarized in Table 4.

### 3.4. Variants of Interest

A total of 93 (strong and very strong) positive samples were successfully sequenced including 61 (65.6%) alpha variants, 8 (8.6%) gamma variants, 22 (23.7%) delta variants and 2 (2.2%) others (not variants of concern). All these samples yielded a positive antigen result on NPS at the previously determined threshold; no variant effect was highlighted.

### 3.5. Modelling the Influence of Epidemic Trends on the Overall Performance of the Assay

**Figure 3:**
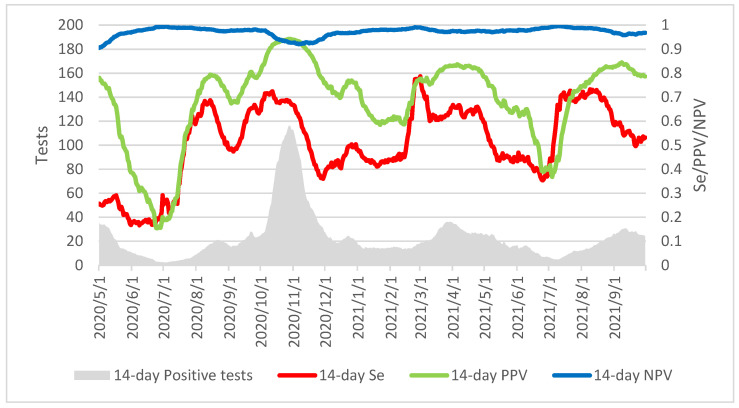
Modelling SARS-CoV-2 automated antigen detection performance on epidemic trends. Data computed from 1 May 2020 to 30 October 2021 using a backward sliding window of 14 days (14-day Se: 14-day sensitivity, 14-day PPV: 14-day Positive Predictive Value, 14-day NPV: 14-day Negative Predictive Value).

The model showed that using the automated SARS-CoV-2 antigen quantification on our day-to-day non-selected routine samples would exhibit important variation of sensitivity and predictive positive value according to the dynamic of the epidemic at that time (Figure 3). Indeed, during low circulation phases, sensitivity decreases sharply due to a very high proportion of weak positive samples. Likewise, a higher proportion of false positive penalizes the positive predictive value. Conversely, during epidemic peaks, the positive predictive value increased above 90% in November 2020 while the negative predictive value decreased slightly below 95% after the end of the first epidemic wave in Belgium in May 2020.

## 4. Discussion

In a previous study [6], we proposed to position the different available assays for the direct diagnostic of COVID-19 in a structured algorithm. Rapid antigen tests were dedicated to the fast diagnostic of symptomatic patients and automated antigen quantification assays were used as a screening method. This strategy allows the provision of a faster and cheaper but reliable alternative to large molecular platforms. In the present study, the Lumipulse^®^ *G* SARS-CoV-2 assay yielded excellent analytical performance on NPS for recently symptomatic patients with a sensitivity of 93.1% for patients with DSO ≤ 7 and a specificity of 99.0%. Compared to our previous study [6], the AUC of the ROC curve improved, which can be attributed to the use of SES instead of an in-house inactivation method but also to the selection of positive samples from patients with DSO ≤ 7. Performances of the Lumipulse^®^ *G* SARS-CoV-2 on NPS were even closer to those of RT-PCR when excluding low positive samples (Se = 96.4%), demonstrating its particular interest to (i) diagnose recently ill patients and (ii) efficiently detect infectious people in a wider screening strategy. As expected, saliva yielded less of a good performance and should be restricted to settings with limited access to swabs or patients presenting contraindication for nasopharyngeal sampling. Comparable results were previously obtained on saliva and on other instruments [11,12,13].

Additionally, we modelled the estimated performance of this automated quantification assay on our day-to-day routine (“all comers” non-selected samples). As expected, in our hospital laboratory an important proportion (46.8%) of samples were weak positive samples, which impaired in this model the overall sensitivity and positive predictive value of the test as compared with results obtained during the analytical evaluation. However, using this extrapolation could be of use to choose the best technique on the best target population in their own setting. Despite this non-selection of samples, the negative predictive value stayed over 95% during the epidemic peaks. This good negative predictive value and the good sensitivity on samples with significant viral load confirm that the use of this technique is interesting for the fast and large screening of asymptomatic patients in contexts such as hospital admission, participation to a social event and travel. Indeed, this technique was implemented at several airports in Germany to screen travelers using oropharyngeal swabs [14], and in Italy in schools using saliva [15]. Only 9.1% (10/110) of the positive samples from patients with DSO ≤ 7 had a weak positive RT-PCR result, whereas it was the case for 29.9% (32/107) of patients with DSO > 7 or unknown. This difference underlines the lower viral concentration at the late infection stage where the infectiousness is lower. The lower performance of automated antigen quantification in this setting should consequently have a limited impact on virus circulation. However, during periods of low virus circulation, this technique should be used in a two-step strategy, with positive samples checked by a reflex RT-PCR, to balance the lower positive predictive value observed during these periods. From a public health perspective, such strategy would dramatically decrease the costs of the screening for a limited risk and free the PCR instruments for more serious cases and other uses.

Automation allows a higher throughput than manual antigen rapid testing (60–120 tests per hour per instrument) and provides an automated, objective reading of the test. Furthermore, the use of UTM also allows secondary PCR checks and sequencing on positive samples.

Our results provide evidence that the Lumipulse^®^ *G* SARS-CoV-2 Ag assay is a robust antigen quantification assay for the detection of SARS-CoV-2 on UTM preserved NPS especially for recently ill patients and people with high viral loads. The use of this technique could offer a fast and cost-efficient solution for diverse situations such as systematic or targeted screening in specific situations (travelers, social events, nightlife etc.).

## Figures and Tables

**Table 1:** Semi-quantification of SARS-CoV-2 RT-PCR results using the Alinity *m* SARS-CoV-2 assay (Abbott Molecular, Des Plaines, IL, USA).

Semi-Quantification	Ct Values	Estimated Viral Load (RNA Copies/mL)
Weak	>29.9	<10^3^
Mild	>23.3–29.9	10^3^–<10^5^
Strong	>16.7–23.3	10^5^–<10^7^
Very strong	≤16.7	≥10^7^

**Table 2:** Study population (N: total number of samples, Ag: antigen quantification, NPS: nasopharyngeal swabs, DSO: days since symptom onset).

Samples Collected	N	Ag (NPS)	Ag (Saliva)
Overall	632	605	144
Negative RT-PCR	408	400	83
Outpatients	304	300	76
Inpatients	104	100	7
Positive RT-PCR	224	205	61
≤7 DSO	116	102	35
>7 DSO or unknown	108	103	26

**Table 3:** Analytical performance of the Lumipulse^®^ *G* SARS-CoV-2 assay on nasopharyngeal swabs (NPS) and saliva vs. RT-PCR on NPS. T: positivity threshold, CI: confidence interval, DSO: days since symptom onset.

Samples Collected	Ag (NPS, T = 2.47 pg/mL)		Ag (Saliva, T = 0.60 pg/mL)	
	N	% (Wilson 95% CI)	N	% (Wilson 95% CI)
Sensitivity (DSO ≤ 7)	95/102	93.1 (86.5–96.6)	25/35	71.4 (54.9–83.7)
Specificity (overall)	396/400	99.0 (97.5–99.6)	82/83	98.8 (93.5–99.8)
Outpatients	298/300	99.3 (97.6–99.8)	75/76	98.7 (92.9–99.8)
Inpatients	98/100	98.0 (93.0–99.4)	7/7	100 (64.6–100)

**Table 4:** Analytical performance of the Lumipulse^®^ *G* SARS-CoV-2 assay on nasopharyngeal swabs (NPS) and saliva vs. RT-PCR on NPS at different levels of viral loads (T: positivity threshold, CI: confidence interval, Ct: cycle threshold value).

Estimated Viral Load (RNA Copies/mL)	Ag (NPS, T = 2.47 pg/mL)		Ag (Saliva, T = 0.60 pg/mL)	
	N	% (Wilson 95% CI)	N	% (Wilson 95% CI)
<10^3^ (Ct > 29.9)	4/38	10.5 (4.2–24.1)	5/8	62.5 (30.6–86.3)
≥10^3^ (Ct ≤ 29.9)	141/167	96.4 (92.4–98.3)	32/46	69.6 (55.2–80.9)
10^3^–<10^5^ (Ct > 23.3–29.9)	36/41	87.8 (74.5–94.7)	8/19	42.1 (23.1–63.7)
10^5^–<10^7^ (Ct > 16.7–23.3)	70/71	98.6 (92.4–99.8)	12/15	80.0 (54.8–93.0)
≥10^7^ (Ct ≤ 16.7)	55/55	100 (93.5–100)	12/12	100 (75.8–100)

## Data Availability

The data presented in this study are available in the Supplementary Material.

## Supplementary Materials

The following supporting information can be downloaded at: https://www.mdpi.com/article/10.3390/diagnostics12020447/s1, Table S1: Dataset.
